# Outcomes after noncardiac surgery in patients with left ventricular assist devices: a systematic review

**DOI:** 10.3389/fcvm.2024.1414444

**Published:** 2024-09-17

**Authors:** Emad Alamouti-Fard, Pankaj Garg, John Yazji, Tara Brigham, Samuel Jacob, Ishaq J. Wadiwala, Si M. Pham

**Affiliations:** ^1^ Cardiothoracic Surgery, Mayo Clinic, Jacksonville, FL, United States; ^2^ Mayo Clinic Libraries, Mayo Clinic, Jacksonville, FL, United States; ^3^ Heart and Lung Transplant National Recovery Program, United Network for Organ Sharing (UNOS), Jacksonville, FL, United States; ^4^ Division of Vascular Surgery, Oregon Health and Sciences University, Portland, OR, United States

**Keywords:** noncardiac surgery, left ventricular assist device, mechanical circulatory support, outcome, complications

## Abstract

**Background:**

The number of patients living with left ventricular assist devices (LVADs) has gradually increased in the past decade. Non-cardiac surgery (NCS) in patients with LVAD poses a unique situation with its inherent challenges.

**Aim:**

We conducted a comprehensive review to investigate the perioperative complications and mortality associated with emergent or elective NCS in patients with LVAD.

**Method:**

A comprehensive literature search for any papers referring to continuous LVAD patients with NCS. All publications with at least five durable LVAD patients who had NCS were eligible for inclusion.

**Result:**

Twenty articles matching our criteria were found and included in our study. This systematic review included 6,476 LVAD patients who underwent 6,824 NCS. There were 5–3,216 LVAD patients with NCS in each study. The median age was between 39 and 65 years, and most of the patients (78.8%) were male. Thirty-day postoperative mortality ranged from 0% to 60%. Eight studies reported no death within the 30 days of the operation. Common complications include gastrointestinal (GI) bleeding, intracranial bleeding, infection, acute kidney injury (AKI), urinary tract infection (UTI), stroke, sepsis, pneumonia, and VAD exchange. Emergent abdominal surgery had the highest (up to 60%) mortality rate, and vascular and neurological operations had the highest complication rates. Due to the diverse range of patients in each publication and the combination of outcomes presented in various publications, a meta-analysis was not conducted.

**Conclusion:**

In LVAD patients, noncardiac surgery may be performed effectively and safely. LVAD patients who undergo non-cardiac surgery may require more transfusions due to their complex coagulopathies. However, perioperative management of LVAD patients undergoing emergent NCS should be optimized to reduce mortality.

**Systematic Review Registration:**

https://osf.io/fetsb/.

## Introduction

Heart failure due to various etiologies (ischemic and non-ischemic heart diseases) affects about 64.34 million people worldwide (8.52 per 1,000) (1) and 6.5 million Americans (2), with an incidence rate of 915,000 cases every year (3). End-stage heart failure is associated with a high mortality rate, and the treatment of choice for heart failure is heart transplantation. However, due to the severe shortage of organ donors, the criteria for selecting heart transplant recipients remain stringent. For patients with heart failure who are not suitable candidates for a heart transplant due to associated co-morbidities, LVAD can reduce mortality and improve 2-year survival significantly compared to maximal guideline-directed medical treatment (GDMT), whose progress has plateaued in recent years (4–6). Due to improvements in survival rates—over 70% for two years and 58.4% for five years with new generation LVADs—and the increasing number of patients undergoing LVAD implantation each year, the population of patients surviving with LVADs is growing rapidly (7). As a result, NCSs in LVAD patients become more common; therefore, clinicians caring for LVAD patients need to be aware of the perioperative management, potential complications, and outcomes of NCSs in these patients.

We limited our systematic review to continuous-flow LVADs since pulsatile LVADs are rarely implanted in contemporary clinical practice.

## Methods

Studies were identified by a librarian developing and running searches in the MEDLINE (1946-Present), Embase (1974-Present), Cochrane Central Register of Controlled Trials (1991-Present), and Cochrane Database of Systematic Review (2005-Present) [all via the Ovid interface], Science Citation Index Expanded (1975-Present) and Emerging Sources Citation Index (2019-Present) [via the Web of Science interface], Scopus [via the Elsevier website interface] (1823-Present), Epistemonikos and the World Health Organizations (WHO) Global Index Medicus databases. Grey literature resources were included in the first search. There were no limits to language or publication date. Search filters to remove animal studies and case reports were used. The search strategies were created using a combination of keywords and standardized index terms. Search terms included MeSH, Embase/ Emtree terms, as well as keywords such as left ventricular assist device, LVAD, and non-cardiac surgical terms, with a particular focus on neurosurgery, abdominal surgery, and metabolic surgery terms. All databases, registers, and grey literature resources were searched on April 13th, 2022, and recently updated to May 31st, 2024. and all papers published until December 31, 2023, were reviewed. Forward citation searching was employed for both searches. A draft search strategy was peer reviewed by a second information professional, John Reynolds. The full search strategies are available here: https://osf.io/fetsb.

All studies with five or more LVAD patients undergoing NCS were eligible for inclusion. Exclusions included perioperative procedures and complications related to index implant hospitalization. Studies containing LVAD-related complications were excluded. Articles were included if more than 80% of the patients had continuous LVAD. The primary endpoint was 30-day postoperative or hospital mortality. Postoperative morbidity and complications, specifically bleeding and device malfunction, were secondary endpoints. All the abstracts without full text were excluded.

## Results

**Figure 1:**
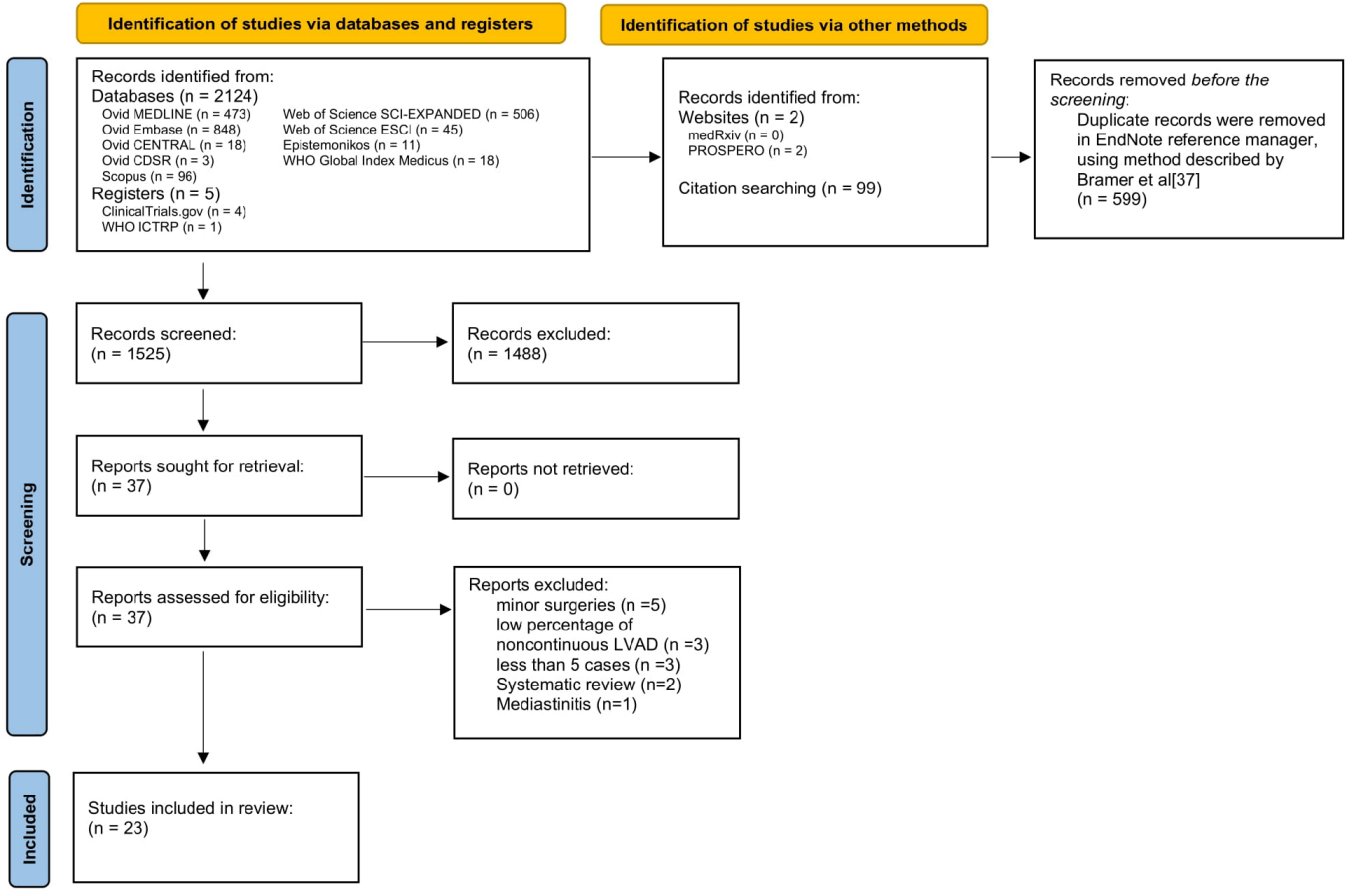
PRISMA flow diagram for literature searches (8).

A systematic review of the databases yielded 2,124 records, 599 of which were duplicates. Initial manual title screening excluded 1,488 off-topic citations. The remaining 37 records were assessed for eligibility, both screener groups identified 37 as meeting the inclusion criteria. Of those, 14 were excluded for the following reasons: minor surgical procedures (5), noncontinuous LVAD (3), series with less than 5 cases (3), systematic review (2), and treatment of mediastinitis (1). (Figure 1).

In the end, 23 papers, with 6,476 LVAD patients who underwent 6,824 NCS, were included in this review. Table 1 summarize the results for restropective studies and large database analyses, repectively. The number of patients in each study ranged from 6 to 3,216 patients. The median ages of the patients in each article were between 39 and 65 years. Seven studies did not distinguish between the bridge to transplant or destination therapy in patients. Thirty-day postoperative mortality ranged from 0% to 60%. Eight studies reported no death within the 30 days of the operation.

**Table 1:** LVAD patient characteristics, type of surgery and outcomes (retrospective studies).

Authors (year)	Total patients	LVAD type	Patients with NCS	Number of NCS	Type of NCS	Mortality (%)		Complications
						Elective	Emergent	
Bauman et al. 2021 (9)	301	HM 2, HM 3	39	39	General	(26%)		N/A
Beetz et al. 2020 (10)	512	350 HVAD, 168 HM2, 53 HM3,	39	39	22 Elective abdominal, 17 Emergency abdominal	0	(47.1%)	Wound infections (25.6%), Postoperative Hemorrhage/Hematoma (43.6%)
Berger et al. 2020 (11)	106	47 HM2, 28 HM3, 31 HVAD	63	105	16 Abdominal; 5 Vascular; 7 Urologic; 9 Neurologic; 8 Orthopedic; 2 Thoracic; 28 Head and neck surgery; 24 ICD/Pacemaker; 5 Ambulant surgery	(3.8%)		N/A
Bhat et al. 2012 (12)	110	HM2	36	63	24 Abdominal; 9 Vascular; 9 Urologic; 7 Oral; 4 Neurologic; 3 Orthopedic; 2 Thoracic; 2 Head and neck surgery; 3 Plastic	(10%)		Intraoperative excess bleeding
Chen et al. 2017 (13)	209	HM2, HVAD, VentrAssist	69	78	55 General; 26 Plastic; 20 Vascular; 10 Urology; 8 Neurologic; 6 Orthopedic; 2 Thoracic	0	(11.6%)	Elective = Infection (8.6%), Bleeding (17.1%), Wound breakdown (5.7%), Others (8.6%); Urgent = Infection (7.0%), Bleeding (11.6%), Others (4.7%)
daSilva-deAbreu et al. 2021 (14)	N/A	4 HM 3, 3 HM2, one HVAD	8	8	Laparoscopic sleeve gastrectomy	0		Post-LSG gastrointestinal bleeding (12.5%); Gastrointestinal hemorrhage (37.5%); Rehospitalization within 30 days after discharge (37.5%)
Gundes et al. 2017 (15)	76	6 HM II, 1 HM III	7	7	Emergency abdominal surgery		(28.6%)	Wound infections (28.5%), Multiple organ failures (28.5%)
Hawkins et al. 2018 (16)	N/A	N/A	11	11	Laparoscopic sleeve gastrectomy	0		Hematemesis (9.0%)
Jeng et al. 2021 (17)	240	3 HVAD, 11 HM2	14	14	Laparoscopic sleeve gastrectomy	0		No complication
Kettyle et al. 2017 (18)	115	21 HVAD, 15 HM2, 1 CentriMag	37	65	21 General, 13 Thoracic, 12 Plastic, 5 Urology, 4 Vascular, 4 ENT, 7 Other services	0		Postoperative bleeding (17%), Postoperative thrombosis (3%), Postoperative infectious complications (14%), UTI (5%), Surgical site infection (5%), Pneumonia (5%)
Morgan et al. 2012 (19)	86	HM2, CF-LVAD	20	25	4 Inguinal hernia repairs, 4 Cholecystectomy, 3 Bilateral salpingo-oophorectomies, 2 Release of SMO, 2 Colon resection, 2 Lipoma excision, 2 Insertion of a tunneled catheter, one Endometrial ablation, one Iliofemoral bypass, one Gastric bypass, one Incision and drainage of knee, one Teeth extraction, one Removal of catheter	0		Blood transfusion of PRBC (36.0%), Surgical exploration (16.0%)
Punchai et al. 2019 (20)	N/A	N/A	7	7	Laparoscopic Sleeve Gastrectomy	0		Arrhythmias, GI bleeding, UTI, Nausea and dehydration, Septicemia due to acute cholecystitis
Rosenberg et al. 2023 (21)	N/A	N/A	5	12	Total joint arthroplasty	0		LVAD thrombus (20%), revision surgery, Infection, Bleeding complications
Rustum et al. 2021 (22)	N/A	30 HVAD,10 HM2, 1 HM3	41	54	Vascular surgery, 22 Emergencies, 32 Elective	(6.30%)	(40.90%)	Prolonged wound healing (16.7%), Perioperative renal insufficiency (51.2%), Hemodialysis (24.4%)
Vigneswaran et al. 2019 (23)	374	12 HM2, 5 HVAD	17	17	Elective laparoscopic: 7 Enteral access placements, 6 Cholecystectomies, 2 Hernia repairs, one Small bowel resection, one Splenectomy	0		N/A
Zenilman et al. 2019 (24)	N/A	5 HM2, 1 HVAD	6	6	Sleeve Gastrectomy	0		Bleeding from the left rectus muscle (16.6%)
Zilbermints et al. 2020 (25)	93	8 HM2, 2 HVAD	10	10	General surgery	0	(60%)	Organ failure (10%); Multiple reoperations (10%)
LVAD patient characteristics, type of surgery and outcomes (Databases studies)								
Briasoulis et al. 2021(NIS USA-2010–2014) (26)	30,323	N/A	3,216	3,216	469 Vascular; 481 Orthopedic; 196 Head & neck; 1,540 General	Vascular (5.3%), Orthopedic (3.1%), Head and neck (23.5%), General (3.8%), Genitourinary (3.2%), Thoracic (11.5%), OBGYN (0%), Neurologic (37.6%) Total (7.7%)		Ischemic stroke (16.6%); Intracranial bleeding (38.3%), GI bleeding (18.7%), Blood transfusion (97.8%), AKI (70.5%), Sepsis (47.2%); Wound infection (13.7%); UTI (26.3%, Pneumonia (34.9%)
McElderry et al. 2022 (Medicare 2012–2019) (27)	14,417	N/A	198	198	186 Gastric sleeve surgery, 12 Gastric bypass surgery	(5.5%)		N/A
Mentias et al. 2020 (Medicare 2012–2017) (28)	8,118	N/A	1,326	1,326	613 General; 219 Thoracic; 199 Orthopedic; 154 Genitourinary; 40 Head and Neck; 101 Vascular	(4.3%)	(10.2%)	Urgent = Ischemic stroke (6.8%), Brain bleed (3.3%), AKI (45.5%), Sepsis (10.3%), AHF (10.1%), Blood transfusion (37.5%); Elective = AKI (25.2%), Sepsis (5.5%), AHF (8.3%), Blood transfusion (23.9%)
Place et al. 2022 (NIS; 2010–2015Q3) (29)	1,805	N/A	290	346	40 BLES, 30 Small bowel resection, 41 Open cholecystectomy, 89 laparoscopic cholecystectomy, 5 Open appendectomy, 31 Laparoscopic appendectomy, 49 open lysis of adhesions, 15 Laparoscopic lysis of adhesions, 36 Open hernia repair, 5 Laparoscopic hernia repair, 5 Laparotomy		(13.8%)	N/A
Sanaiha et al. 2019 (NIS USA-2005–2015) (30)	23,441	N/A	719	719	Emergency abdominal surgery		(41.5%)	GI Obstruction, GI ischemia, GI bleeding, pancreatitis, Hernia, Cholecystitis
Taghavi et al. 2016 (NIS 2007–2010) (31)	1,397	N/A	289	459	153 General, 141Thoracic, 140 Extremity/vascular surgery, 16 Oral/maxillofacial surgery, Orthopedic procedure = <10, Urological surgery = <10	(22.8%)		Wound infection (9.1%), Pneumonia (18.5%), UTI (14.1%), Bleeding complication (44%), AKI (53.4%), Pulmonary embolus/DVT (4%), Sepsis (19.1%), Any complication (87.2%)

HM2, heartmate II; HVAD, heart-ware ventricular assist device; HM3, heartmate III; AKI, acute kidney injury; AHF, acute heart failure; GIB, gastrointestinal bleeding; UTI, urinary tract infection; SMO, small bowel obstruction; N/A, not applicable; AKI, acute kidney injury; AHF, acute heart failure; GIB, gastrointestinal bleeding; UTI, urinary tract infection; SMO, small bowel obstruction; NIS, National Inpatient Sample database; N/A, not applicable.

Due to the wide heterogenicity in reports of the outcomes of the studies, we decided not to perform a meta-analysis or data synthesis. LVAD types, NCS, and perioperative anticoagulation management are among the variables reported with significant inconsistency, which could have reduced the validity of the formal analysis.

Because non-cardiac surgical procedures in LVAD comprise of a heterogenous group of patients, we categorized these procedures into general and abdominal surgeries, bariatric surgery, vascular surgery, and neurosurgery.

General surgical operations in LVAD patients range from superficial procedures on the skin and soft tissues to intra-abdominal procedures via laparoscopic or open operations (10, 14–17, 20, 23–25).

We found 21 out of 23 publications that reported the results in 4,151 general surgical procedures, with 244 gastric bypass operations that were discussed exclusively in 6 publications; therefore, we will discuss the latter operations separately in the bariatric surgery section. Some citations did not report data regarding different types of surgery, while some publications reported the mortality and complication in different kinds of operations. The mortality rates vary from 0% to 60% for bariatric operations (14, 16, 17, 20, 24) and emergency abdominal surgeries (25), respectively. More details about mortality rates and complications are available in Tables 1, 2. Next, we will discuss the outcomes of different non-cardiac operations in LVAD patients.

**Table 2:** Mortality and morbidities of non-cardiac surgery in LVAD patients.

Type of surgery	30 days mortality rate	Complications	References
Bariatric surgery	(0%–5.5%)	GI Bleeding, driveline infection, UTI, septicemia, bleeding into the rectus muscle	(14, 16, 17, 20, 24, 29)
General surgery	Elective = (0%–3.8%) Emergent = (28.6%–60%)	VAD exchange due to thrombosis and infection, GI bleeding (6.4%), AKI (24.6%), UTI (7.1%), sepsis (10.3%), pneumonia (4.4%)	(10, 15, 25, 26)
Vascular	Elective = (5.3%–26.8%) Emergent = (40.9%)	GI bleeding (12.6%), AKI (26.4%), Sepsis (9.4%), UTI (8.3%), pneumonia (3.2%)	(22, 26)
Neurosurgery	(25%–50%)	Stroke, GI bleeding (4.2%), AKI (11.8%), sepsis (9.4%), pneumonia (16.5%)	(11–13, 26)
Orthopedic surgery	(0%–3.1%)	GI bleeding (6.4%), AKI (18.7%), sepsis (8.3%), UTI (5.2%), pneumonia (3.1%)	(21, 26)
Head and neck	In-hospital mortality (23.5%)	GI bleeding (8.2%), AKI (41.3%), UTI (15.8%), pneumonia (26%)	(26)

AKI, acute kidney injury; GI Bleeding, gastrointestinal bleeding; UTI, urinary tract infection; VAD, ventricular assist device.

## Discussions

### General and abdominal surgeries

A study by Beets et al. focused on the outcome of abdominal surgery in LVAD patients. In their study, a total of 604 patients with LVAD from a single center were included, out of which 39 (6.5%) patients underwent abdominal surgery. A total of 22 patients (56.4%) underwent elective abdominal surgeries, including abdominal wall hernia repairs, partial colectomies, and cholecystectomies. There was no operative or postoperative death in patients undergoing elective abdominal surgery at a median follow-up of 23 months (1–78 months). A total of 17 patients (43.6%) underwent emergency surgery. The most common indications for emergency surgery were intestinal ischemia or perforation. In the first 30 days after abdominal surgery, eight patients (47.1%) died from sepsis and subsequent multiple organ failure, resulting in a dismal median survival rate of one month. Patients who underwent abdominal surgery tended to have significantly lower rates of a subsequent heart transplant and had a higher rate of VAD exchange due to thromboses or infections before or after the abdominal surgery (10). Another study, by Gundes et al., consists of 76 patients with LVAD implantation, seven patients underwent emergent laparotomy at 79.1 ± 79.4 days after LVAD implantation. Indication for emergent laparotomy in their series was abdominal compartment syndrome related to retroperitoneal hematoma (2), ileus (1), iatrogenic splenic injury (1), splenic abscess (1), acute abdomen (1), and pelvic abscess related to stump leakage in a patient with rectal cancer operation (1). Perioperative mortality was (28.6%) (15). The drivelines were removed from the lower right quadrant in 6 patients and the left lower quadrant in 1 patient.

For laparoscopic surgery, Vigneswaran et al. (10), reported the outcomes 17 patients who underwent a elective laparoscopic procedures: enteral access placement (7), cholecystectomy (6), hernia repair (2), small bowel resection (1) and splenectomy (1), there was no operative or postoperative mortality, LVAD related complication, or need for conversion to open or reoperation. No patient had thrombotic events, bleeding, or complications necessitating withholding anticoagulation. However, 5 of the 17 patients required intraoperative blood transfusion. These authors stated that they used the open periumbilical Hasson technique for port placement.

Place et al. conducted a study utilizing data from the National Inpatient Sample covering the period from 2010 to the third quarter of 2015. Their findings indicated a mortality rate of 13.8% for abdominal surgeries in LVAD patients (29).

Results from these studies reiterate that patients with LVAD can safely undergo elective abdominal procedures (23). However, every case needs to be well-planned by a multidisciplinary team, including general surgeons, heart failure cardiologists, cardiac surgeons, hematologists, anesthetists, and intensivists. Although the presence of LVAD does not preclude the patient from undergoing laparoscopic abdominal operations, a surgeon needs to be cognizant of bleeding and thrombotic risks, port placement sites, and perioperative management related to LVAD and anticoagulation.

### Bariatric surgery

Many patients with heart failure suffer from morbid obesity, a contraindication for a heart transplant due to poor outcomes. It is advised that patients with a BMI above 35 kg/m^2^ receive counseling to decrease their BMI to below 35 kg/m^2^ before being listed for transplantation (32). However, limited physical activity due to heart failure makes it nearly impossible for the patient with heart failure to lose weight. Therefore, bariatric surgery has become a bridge to heart transplants in these patients. In addition, bariatric surgery has been shown to lower BMI, reduce heart failure symptoms, and enhance the overall quality of life (33).

Furthermore, with the widespread use of LVADs in the treatment of HF in patients with obesity, evidence of the efficacy of bariatric surgery in patients with severe HF and LVAD is growing (34, 35). In this systematic review, 46 patients (26 male and 20 female) underwent bariatric surgery in retrospective studies (14, 16, 17, 20, 24), and one study was conducted using the Medicare database. In retrospective studies, laparoscopic sleeve gastrectomy was the procedure that was performed the most. The procedure was associated with an excellent outcome with no perioperative or one-year mortality. There were only two (4.35%) deaths at a follow-up of two years. The most common complication in these patients was gastrointestinal bleeding. Other less common complications were urinary tract infections and arrhythmia due to hypokalemia. One patient required readmission within 30 days due to hematemesis.

McElderry et al. conducted a study using data from the Medicare database spanning from 2012 to 2019. The study focused on LVAD patients who underwent either gastric sleeve surgery or gastric bypass surgery. They reported a mortality rate of (5.5%) for these procedures (27).

The results of these studies show that bariatric surgery is a safe and viable option for morbidly obese patients with LVAD to lose weight. Hopefully, with new weight-loss medications becoming more widely available, the need for bariatric surgery in these patients becomes less (36).

### Urgent vs. elective abdominal surgeries

There is a significant difference in outcomes in urgent vs. elective abdominal surgeries. As expected, elective surgeries have lower mortality and infection rates in LVAD patients (25). Chen et al., reported that the risk of infection and bleeding in elective operations is not higher than in urgent surgeries. However, emergent surgeries have a higher infection rate and higher mortality (13). There were no statistically significant differences among the groups in terms of postoperative ICU length of stay (LOS), postoperative length of time on the ventilator, hospital LOS, or postoperative thrombotic or bleeding complications (18). All groups, whether elective, urgent, or emergent, have higher early and late mortality rates comparing LVAD patients without NCS (28).

Sanaiha et al. showed that cachexia could increase the rates of emergency general surgeries in LVAD and heart transplant patients. LVAD patients with emergency surgery have higher mortality, hospitalization costs, and lengths of stay after adjusting for their comorbidities than non-emergency patients. In subgroup analysis, these authors reported that patients who required small bowel resection had the highest mortality rates, although wide confidence intervals limit the strength of the results (30).

### Vascular surgery

There were 9 out of 20 articles that reported on outcome of 805 vascular procedures. Some reported specific results on vascular surgery, but others reported the outcomes along with other general surgeries. Two articles reported their experience in vascular surgery separately (22, 26).

Vascular surgery in LVAD patients carries a mortality rate of 5.3% (in hospital) (26) to 26.8% (30 days mortality) (22), and emergent procedures have higher mortality rates than elective ones (40.9% vs. 5.3%). Vascular surgeries in LVAD patients have a high ischemic stroke and gastrointestinal bleeding rates, 10.7% and 12.6%, respectively (22, 26).

### Neurosurgery

Neurosurgical procedures in LVAD patients have the highest complication rate. LVAD patients required a craniotomy for an intracranial hemorrhage carry a 25%–50% mortality (11–13, 26). Most patients required blood product transfusions due to excessive blood loss during the procedure (12, 26). About 30%–40% of LVAD patients with neurosurgery have other complications, including acute kidney injury or sepsis, while 10% had a stroke after the neurosurgery. (26) Among non-cardiac surgeries, neurosurgery has the highest mortality rate in LAVD patients (12).

## Conclusion

Patients with continuous-flow LVADs who had noncardiac surgeries were reviewed in this systematic study. Noncardiac surgeries can be performed effectively and safely on LVAD patients. Morbidity and mortality were caused by patient's conditions, complications such as bleeding, stroke, acute kidney injury, infections, and many others. The care for these complex patients requires multiple expertises and many resources. A multidisciplinary approach at tertiary centers with healthcare providers who have expertise in LVADs can significantly improve postoperative outcomes of these patients.

Our protocol for managing non-cardiac surgery (NCS) in patients with left ventricular assist devices (LVADs) is tailored based on the type of surgery, the specific LVAD model, and the patient's condition. Generally, the following steps are undertaken:

1. Preoperative:
  Consultation with heart failure cardiologists, cardiac surgeons with expertise in LVAD, cardiac anesthesiologists, and potentially a hematologist.
  Decisions regarding the reversal and bridging of anticoagulation therapy depend on whether the surgery is urgent or elective.
2. Intraoperative:
  Implementation of hemodynamic monitoring
  Meticulous surgical techniques to achieve hemostasis
  Administration of blood products to help with hemostasis if needed.
3. Postoperative:
  Continuation of hemodynamic monitoring as needed.
  Transitioning the patient back to oral anticoagulation therapy.
  Close monitoring for wound infection, stroke, and LVAD function.

By following this protocol, we aim to optimize patient outcomes and ensure the safety and efficacy of NCS in LVAD patients.

## Limitations

Our study faced several limitations. All of the studies were retrospective in natures, and due to the data heterogeneity, we could not perform a meta-analysis. Additionally, the complications and mortality rates were not specified for the type of LVAD, preventing us from comparing the different LVAD types.

## Data Availability

The original contributions presented in the study are included in the article/Supplementary Material, further inquiries can be directed to the corresponding author.
